# Induction of MAP Kinase Homologues during Growth and Morphogenetic Development of Karnal Bunt (*Tilletia indica*) under the Influence of Host Factor(s) from Wheat Spikes

**DOI:** 10.1100/2012/539583

**Published:** 2012-04-01

**Authors:** Atul K. Gupta, J. M. Seneviratne, G. K. Joshi, Anil Kumar

**Affiliations:** ^1^Department of Molecular Biology and Genetic Engineering, College of Basic Sciences and Humanities, GBPant University of Agriculture and Technology, Pantnagar 263 145, India; ^2^Department of Biotechnology, HNB Garhwal University, Srinagar, Uttarakhand 246174, India

## Abstract

Signaling pathways that activate different mitogen-activated protein kinases (MAPKs) in response to certain environmental conditions, play important role in mating type switching (Fus3) and pathogenicity (Pmk1) in many fungi. In order to determine the roles of such regulatory genes in *Tilletia indica*, the causal pathogen of Karnal bunt (KB) of wheat, semi-quantitative and quantitative RT-PCR was carried out to isolate and determine the expression of MAP kinase homologues during fungal growth and development under *in vitro* culture. Maximum expression of TiFus3 and TiPmk1 genes were observed at 14th and 21st days of culture and decreased thereafter. To investigate whether the fungus alters the expression levels of same kinases upon interaction with plants, cultures were treated with 1% of host factors (extracted from S-2 stage of wheat spikes). Such treatment induced the expression of MAPks in time dependent manner compared to the absence of host factors. These results suggest that host factor(s) provide certain signal(s) which activate TiFus3 and TiPmk1 during morphogenetic development of *T. indica*. The results also provides a clue about the role of host factors in enhancing the disease potential due to induction of MAP kinases involved in fungal development and pathogenecity.

## 1. Introduction

Karnal bunt (KB) caused by *Tilletia indica *(*Neovosia indica Syn.*) is a fungal disease that affects wheat, durum wheat and triticale. KB is an emerging infectious disease (EID) with profound socioeconomic implications [1]. It is a seed-borne disease which typically causes partial conversion of individual kernels into sori filled with fetid teliospores thus affecting yield and quality [2]. *T. indica* is a dimorphic pathogen occurring in haploid (mycelia and sporidia) and diploid (teliospore) stages of its life cycle. It is of particular interest because it is a representative of smut fungi that only cause infection on florets during anthesis. Infection occurs after heading when sporidia produced from teliospores at the soil surface are dispersed to the glumes of the wheat spike. Fungus threads (hyphae) from sporidia penetrate stomata and grow intercellularly to the base of the developing kernel. Commonly, only some kernels in a spike are affected. The fungus infects one or more developing seed on a head, but usually not all the seeds [3]. As so far, none of the control measures had been proven to be satisfactory for the disease management, hence it becomes inevitable to understand the biology of the pathogenic fungus through dissecting the complex signaling pathways involved in fungal development and pahogenecity [4].

The disease attack at different plant developmental stages has been reported during specific growth stages ranging from boot swelling to partial emergence of spike [5, 6], boot stage to complete emergence of spike [7], early boot stage to anthesis [8] between spike emergence and anthesis [9, 10], or during anthesis [6, 11, 12]. The disease presence is clearly noticed in spike when ear head is just peeping out (*Z* = 58, S2) from the tip compared to the leaves (*Z* = 16, Sv), boot stage (*Z* = 46, S1), ear head completely out from the boot leaves/seed formation stage (*Z* = 77, S3) [13]. Thus, plant development stages reported susceptible to infection vary considerably. Since there is a close association between the host developmental stages and infection of Karnal bunt, it is important to study the development of disease and the role of host factors in processes ranging from flowering to seed development and subsequently their effect on the developmental stages of *T. indica.* In our laboratory, elaborative efforts have been made to study the morphogenetic development of the fungus in the presence of host extracts prepared from different parts of wheat plant. The aqueous, salt, methanol, and acetone extracts of stem, leaf, and S-1, S-2, and S-3 stages of inflorescence exhibited differential growth in terms of radial growth and mycelial biomass. Maximum growth was seen in acetone extracts of S-2 stage of inflorescence, which induced mycelial growth due to involvement of MAP kinase machinery which is required for controlling processes critical for development of disease [14, 15], and key stages of morphogenetic development after the perception of an external stimulus [16]. 

 A fundamental property of living cells is the ability to sense and respond appropriately to changing environmental conditions and various other stimuli. One frequently utilized molecular device for eliciting these responses is the three-tiered cascade of protein kinases known as the mitogen-activateed protein kinase (MAPK) module [17]. Mitogen-activated protein (MAP) kinase signaling pathways are ubiquitous and evolutionarily conserved in eukaryotic organisms connecting cell surface receptors to critical regulatory targets within cells that result in various morphogenetic processes [18]. MAP kinase activity is regulated through a tiered cascade composed of a MAP kinase, a MAP kinase kinase (MEK), and a MAP kinase kinase kinase (MEKK), first recognized in *Saccharomyces cerevisiae*. In *Saccharomyces cerevisiae,* 5 MAP kinase pathways have been identified [19]. These enzymes are regulated by characteristic phosphorelay system in which a series of three protein kinases phosphorylate activating one and another. Intercellular targets are subsequently regulated by phosphorylation and include transcription factors and cytoskeletal proteins [20].

In other fungus *Magnaporthe grisea*, a well-conserved MAP kinase gene Pmk1 is essential for fungal pathogenesis and for production of female reproductive structures [21]. Unlike the situation in fungal-plant pathogens, the Pmk1-like MAPK pathway is not required for virulence in the fungal-fungal interaction [22]. Further studies have revealed how protein orthologous to Pmk1p/Fus3p/Kss1p are required for pathogenecity in many other phyto-pathogenic fungi [23]. The Fus3 MAP kinase pathway controls the transduction of the pheromone signal and is activated in response to the binding of a peptide-mating pheromone to cell type-specific pheromone receptors. In addition to activating transcription, transduction of the mating response results in reorientation of the cytoskeleton and secretary apparatus to polarize toward a mating partner [24]. Cyclic AMP and MAPK signaling are involved in this process. In the phytopathogenic fungus *Ustilago maydis*, pheromonemediated cell fusion is a prerequisite for the generation of the infectious dikaryon. The pheromone signal elevates transcription of the pheromone genes and elicits the formation of conjugation hyphae.

Dibutyryl c-AMP, an analogue of c-AMP, induces sporidia formation in *T. indica*. The fungus on exposure to dbc-AMP experienced morphological differentiation from vegetative mycelial phase to sporogenous mycelial phase and was induced to produce filiform sporidia [25] through involvement of MAP kinase module(s) in such morpho-genetic transition and development. However, so far no orthologous genes/proteins of mitogen signaling pathway have been identified in KB pathogen of wheat which enter the plant through stomata and grow through intercallinary division unlike other *Magnaportha grisea* which enter the plant by direct penetration through formation of specialized infection structures in the form of appressoria. Taking the advantages of evolutionarily conserved MAP kinase signal transduction pathways for regulating critical processes of disease development in diverse pathogenic fungi even distantly related with very different modes of infection, two homologues of MAP kinase (Fus3 and Pmk1) from KB were identified in our laboratory by PCR-based approaches. In order to establish the molecular basis of induced mycelination in presence of host factor(s) derived from wheat spikes and pathogenecity, in the present study, attempts were made to study the expression of TiFus3 and TiPmk1 MAP kinase genes under the influence of host factors and its relation with morphogenetic development and pathogenesis in *T. indica.*

## 2. Materials and Methods

### 2.1. Collection of Seeds

The resistant (HD29) and susceptible wheat varieties (WH542) were collected from Punjab Agriculture University, Ludhiana. The resistant genotype (HD29) was developed through conventional breeding approach and showed resistance against Karnal bunt as evident by pathogen inoculation studies.

### 2.2. Preparation of Host Factor(s)

Acetone extracts were prepared from spike tissues collected from susceptible (WH542) wheat varieties in boot emergence stage (S2). Using pestle and mortar, wheat spikes (50 g) were ground in liquid nitrogen to a fine powder. Finely ground plant tissues suspended in cold acetone, in the ratio of 1 g of sample in 10 mL of acetone. The suspension was then agitated in cold condition for 5 hours and filtered through muslin cloth to remove larger debris and stored at 4°C in tightly capped bottles. Before starting the experiments, acetone was evaporated at room temperature using flash evaporator or blowing hot air over the solution. Dried material obtained was resuspended in 1/10th of the volume of sterilized distilled water and filtered through 0.22 *μ* filter before incorporation into the culture media as host factors [14].

### 2.3. Fungal Culture and Harvesting of Mycelium

The fungus *T. indica* was cultured in modified potato dextrose liquid media. All the constituents of the potato dextrose media were dissolved in distilled water. 100 mL of liquid media was transferred to 250 mL conical flasks and autoclaved. Then these flasks were inoculated with mycelial discs or loop-full of inoculums from the slants prepared earlier. The cultures were incubated in BOD incubator at 22 ± 2°C under light and dark conditions. The growing liquid cultures of *T. indica* were harvested at the 7th, 14th, 21st, and 30th days with and without host factor(s). The media containing the mycelial mat of *T. indica* was filtered through a folded muslin cloth and washed several times in PBS (0.05 M, pH 7.2) followed by sterilized distilled water. The wet mycelial biomass and total soluble protein extracted from fungal cultures (grown in presence and absence of host factors) at different intervals were determined for plotting the growth curves. The concentration of soluble protein of mycelia was determined by Bradford method [26]. The wet mycelia were lyophilized for 5 hours to obtain the dry weight. Dry mycelial masses were stored in −80°C for subsequent expression studies.

### 2.4. Morphological Observation and Sporidial Count

The fungal cultures were stained with cotton blue and observed under light microscopy. The formation of sporidia in fungal cultures grown on solid PDA medium at different time intervals of growth and development of *T. indica* was calculated using haemocytometer. 10 mL of sterile distilled water was added to petri plate and gently moved back and forth and water containing sporidia was decanted to sterilized oak-ridge tubes and centrifuged at 4000 rpm for 10 min to get sporidial pellet. Supernatant was discarded and the pellet was again washed in 1 mL sterile distilled water and centrifuged for 15 minutes at 3000 rpm. The pelleted sporidia were finally dissolved in 1 mL sterile distilled water. The sporidia collected were enumerated with the help of hamocytometer under microscope.

### 2.5. Pathogenicity Testing

Disease scoring is primarily based on the percentage of infected kernels. Surface-sterilized wheat seeds (*Triticum aestivum* cv. WH542 and HD29 susceptible and resistant, resp.) were germinated on wet paper and were planted on commercial soil mix. Plants were grown at 22°C/18°C (12 h light/12 h dark) in a glass house. The pot experiment was laid out in a randomized block design having different treatment (control (C), pathogen inoculation (P), and host factor(s) treated pathogen inoculation (HFP)) in both susceptible and resistant wheat cultivars having five replicates and designated as SC, SP, SHFP and RC, RP, RHFP, respectively. The injection technique was adopted in which the inoculums are injected with a hypodermic syringe into the boot just as awns emerged [27, 28]. High percentage of infection can be obtained with this technique. Five ear heads were artificially inoculated using hypodermic syringe with the 21-day-old sporidial cultures (10^6^ sporidia/mL) in the month of January. Inoculated ear heads were covered with butter paper to prevent natural infection as well as to maintain moisture. Average percent-infected grains were calculated after harvesting in three successive years.

### 2.6. Disease Scoring

 Disease scoring was done on the basis of the percentage of infected kernels. Average percent-infected grains were calculated after harvesting. As most of the bunted grains were partially infected, numerical values, depending upon the extent of damage to the grains, were given for calculating coefficient of infection. Number of grains showing incipient infection, blackening extended up to half of the grain, 3/4 of grain and infected grains were multiplied with the numerical values 0.25, 0.5, 0.75, and 1.0, respectively, and then divided by 100 to obtain percent coefficient of infection [29].

### 2.7. Preparation of Total RNA and cDNA Synthesis

Total RNA was isolated from each sample by using one-step RNA isolation reagent (Trizole) from Bio Basic Inc., according to the manufacturer's instructions. RNA preparations were subjected to DNase digestion according to manufacturer's instruction (Fermentas International Inc., Canada). Total RNA (5 *μ*g) of each sample was used to synthesize first-strand cDNA by using oligo(dT)_18_ primer with RevertAid H Minus M-MuLV Reverse Transcriptase (RT) (Fermentas International Inc., Canada) according to the manufacture's instruction. The efficiency of cDNA synthesis was assessed by reverse transcriptase PCR amplification of a basal/housekeeping transcript, for example, ribosomal protein S17 (RPS17).

### 2.8. *In Silico* Sequence Analysis

**Figure 1:**
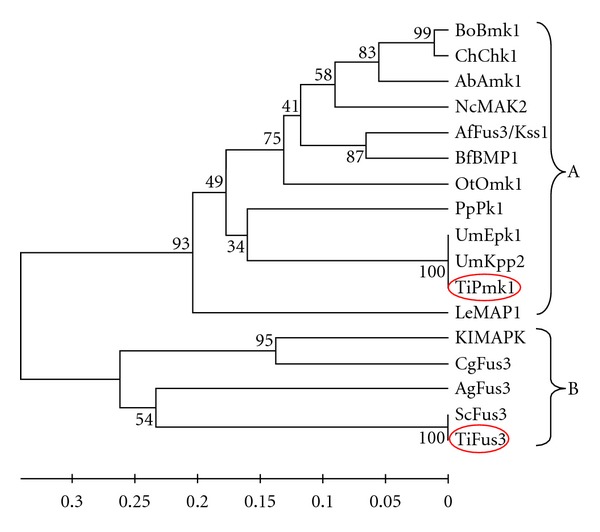
Phylogenetic analysis with TiFus3, TiPmk1, and selected fungal MAP kinases (GenBank accession numbers in parenthesis). *Bipolaris oryzae, *BoBmk1 (AB180104.1);* Cochliobolus heterostrophus, *ChChk1 (AF178977.1);* Alternaria brassicicola, *AbAmk1 (AY515257.1);* Neurospora crassa, *NcMAK2 (AF348490.1); *Aspergillus flavus,* AfFus3/Kss1 (XM002374886.1);* Botryotinia fuckeliana, *BfBMP1 (AF205375.1);* Olpitrichum tenellum, *OtOmk1 (EU479712.1);* Postia placenta, *PpPk1 (XM002471663.1);* Ustilago maydis, *UmEpk1 (XM754359.1), UmKpp2 (AF193614.1);* Tilletia indica,* TiPmk1 (FJ571362.1), TiFus3 (HQ268553);* Lentinula edodes, *LeMAP1 (AB446447);* Kluyveromyces lactis, *KlMAPK (XM454426.1); *Candida glabrata, *CgFus3 (XM447892.1);* Ashbya goesypii *AgFus3 (NM210920.1);* Saccharomyces cerevisiae *ScFus3 (NM001178256.1). The phylogram was constructed by UPGMA method of MEGA version 4.0.02. Bootstrap values are indicated against each branch.

*In silico* analysis were performed in order to confirm the MAP kinase sequences from *T. indica *and their relatedness. The homology search of the Fus3 and Pmk1 was done through Blast search tool of NCBI (http://www.ncbi.nlm.nih.gov) using Blastn and tBlastx algorithm. All sequences (Figure 1) from different fungi were aligned using ClustalW method after that phylogenetic tree was constructed using UPGMA method of MEGA version 4.0.02 [30]. Each node was tested using the bootstrap approach by taking 1,000 replications and a random seeding of 24,054 to ascertain the reliability of nodes. The number is indicated in percentages against each node. The branch lengths are drawn to scale indicated. 

### 2.9. Semiquantitative RT PCR Analysis

To analyze the MAPKs (TiFus3 and TiPmk1) transcript levels in *T. indica* culture grown in presence and absence of host factors at different time intervals, MAPKs (TiFus3 and TiPmk1) specific primers were designed from first-time cloned, sequenced and nucleotide sequences of TiFus3 and TiPmk1 that were submitted as partial DNA fragments of 300 kb and 223 kb from our laboratory to NCBI database with accession numbers as HQ268553 and FJ571362.1, respectively. The gene-specific primers for the expression analysis by RT-PCR and qRT-PCR were used TiFus3 Fwd ACAATTCAGAGCCCACAGGT & Rev ATCTCTGCCAGGGAAGATTG and TiPmk1 Fwd CCGATGACCACTGTCAGTACTTT & Rev CAACGTATTCGGTCATGAAACC which yield the product size of 180 and 210 bp, respectively. Ribosomal protein S17 (RPS17) gene was selected as endogenous internal standard, because it is a house-keeping gene and expressed constantly. The RPS17 primer used as internal control forward 5′-CGA ACC AAG ACG GTG AAG AAG-3′ Reverse 5′-CCT GCA ACT TGA TGG AGA TAC C-3′. cDNAs were exponentially amplified using (Fermentas International Inc., Canada) Taq Polymerase. PCR was performed in 25 *μ*L of 1x KCl buffer (Fermentas International Inc., Canada) containing 0.2 mM dNTPs, 30 ng of each primer, 1.5 mM MgCl_2_, 0.8 U Taq DNA polymerase (Fermentas International Inc., Canada), and 100 ng of cDNA. Amplification was carried out according to the following temperature profile: 4 min initial denaturation at 94°C; 30 cycles of 94°C for 30 second; 57°C for 20–30 second, 72°C for 45 second; final extension of 5 min at 72°C; final hold at 4°C. Densitometry analysis was done with the help of Gene Profiler software, Alpha Innotech Corporation USA. Briefly, individual gels were scored by placing the curser over individual band and recording the relative densitometry values of three independent gels used for expression analysis.

### 2.10. Quantitative Real-Time PCR

Real-time PCR was done in triplicate reaction of three different cDNAs prepared from three RNA samples isolated separately at different time intervals in presence and absence of host factor(s) using the 5 Prime Real Master Mix SYBR ROX (Eppendorf India Ltd.) according to manufacturer's instructions. The 5 Prime uses the fluorescent dye, SYBR green, to detect PCR products. The thermocycler used was eppendorf thermocycler ep realplex. Two-step real time PCR was carried out using cDNA prepared as mentioned earlier from different time interval in presence and absence of host factors. The primers for MAPKs and RPS17 genes used were the same as used for semiquantitative RT-PCR analysis. The reverse transcription efficiencies of MAPKs and RPS17 genes were almost equal as analyzed by comparing the *C*_*T*_ values at different dilutions of cDNA [31]. Final concentrations, in a total volume of 20 *μ*L, were 2.5x Real Master Mix SYBR ROX/20x SYBR solution, 100 nM of each forward and reverse primers, and 100 ng of cDNA. The following amplification program was used: 95°C for 2 min, 40 cycles of 95°C for 15 sec, followed by 1 minute at 60°C. All samples were amplified in triplicate and the mean value was considered. Completely randomized design (CRD) was used for analyzing the gel data and real-time data. The cycle threshold (Ct) value is the number of cycles required to accumulate enough SYBR green fluorescent signal to exceed the threshold (background) level. The Ct value is proportional to the amount of RT-PCR product and was used for quantification. The relative value obtained for quantitation was expressed at 2_*T*_^−ΔΔ*C*^, where Δ*C*_*T*_ represents the difference between the *C*_*T*_ value of the sample and that of RPS17 (endogenous control) in the same sample and ΔΔ*C*_*T*_ is the difference between the Δ*C*_*T*_ value of a sample and that of its respective control.

### 2.11. Statistical Analysis

Three independent determinations for disease scoring, time-dependent expression of MAPKs homologues (TiFus3 and TiPmk1 genes) in absence and presence of host factors, were taken and mean ± SE values were calculated for statistically analysis using paired *t*-test and GraphPad Prism 5.04 software.

## 3. Results and Discussion

Mitogen-activated signal transduction pathways play a crucial role [32, 33] in development of virulence levels in pathogens. Through MAPK pathways, pathogens respond to external stimuli and alter their own features such as cell wall integrity, mating, morphological transition, and adaptation to stress factors and this modification leads to generate different virulence levels in phyto pathogens [24]. Homologous of several MAPKKK, MAPKK, and MAPK have been characterized in several pathogenic filamentous ascomycetes including wheat pathogen and play key roles in infection structure (appressorium) formation and host colonization [15, 34]. The KB is slow growing fungal pathogen and its development is concomitantly dependent on the host's flowering to grain filling stages. The boot emergence stage of developing spikes at anthesis is the most susceptible stage and disease infectivity significantly drops when ear head is completely out from boot leaf and at the postanthesis stage. Hence, it is quite worthwhile to study the influence of host factor(s) on the expression of fungal MAPKs genes in order to simulate the fungal growth and morphogenetic development under *in vitro *cell culture by mimicking some of the microenvironment of anthesis (S-2) stage of developing spikes.

### 3.1. Pathogenicity Testing of Cultivars after Host Factor(s) Treated Sporidial Suspension Inoculation

The most favorable weather for KB infection coincides with wheat heading. It was demonstrated that infection occurred most reliably after hypodermically injecting a suspension of sporidia into the wheat boot at the awns emerging stage when the ear head just peeps out at the tip or from center [35]. Percentage disease severity of this stage is 22.2 as compared to 6.97 of boot stage and 6.9 of ear head half outside boot leaf [36]. Therefore, in order to check whether host factor(s) induce disease severity, host factor(s) pretreatment was done at the time of KB culturing prior to artificial inoculation of the KB pathogen in wheat spikes of both varieties when the ear head just peeped out of the boot. Results of infectivity tests carried out by boot injection are presented in Table 1.

On comparing the pathogen-inoculated plants of both varieties (RC and SC), the values of % infection, coefficient of infection, and overall response of susceptible variety were found to be greater than the resistant variety. However, on host factor(s), application values of % infections were increased in both susceptible and resistant varieties, showing induction of infection. Host factor(s) mediated induction was found to be more pronounced in susceptible variety than the resistant variety. It was observed that host factor(s) significantly (*P* < 0.05) changed the overall response value toward pathogen from 34.69 to 136.51 in susceptible (SP versus SHFP) and from 10.59 to 20.51 in resistant variety (RP versus RHFP). The coefficient of infection (CI) was also high in the presence of host factor(s) in pathogen-inoculated varieties from 0.07 to 0.12 in case of susceptible variety and from 0.04 to 0.05 in case of resistant variety. The role of host factor(s) in increasing disease incidence also became evident from observation of seeds harvested from both varieties. It was found that host factor(s) considerably increase the amount of seed blackening in wheat seeds after pathogen inoculation in both varieties. This clearly suggests that host factor(s) can elicit the signal responses in favor of the pathogen to enable it to increase KB pathogenesis. This indicates the causal link between host factor(s) and disease progression only through modulation of fungal development probably through alteration of MAP kinase machinery responsible for fungal development and virulence.

### 3.2. Sequence Analysis

Phylogenetic tree of TiPmk1 and TiFus3 was constructed taking genes from *Bipolaris oryzae*, *Cochliobolus heterostrophus*, *Alternaria brassicicola*, *Neurospora crassa*, *Gibberella zeae*, *Aspergillus flavus*, *Botryotinia fuckeliana*, *Olpitrichum tenellum*, *Postia placenta*, *Ustilago maydis*, *Lentinula edodes*, *Kluyveromyces lactis*, *Candida glabrata*, *Ashbya goesypii*, and* Saccharomyces cerevisiae* by UPGMA method of MEGA version 4.0.02 which showed two major clusters A and B as shown in Figure 1. Cluster-A contains both Pmk1 and Fus3 genes of different fungi while Cluster-B contains Fus3 gene of different fungi. Pmk1 genes of* T. indica* and *U. maydis*—both belonging to Basidomyceates class—are present in same Cluster-A while Fus3 genes of *T. indica* and *S. cerevisiae* are present in same Cluster-B. The *in silico* phylogenetic study clearly reveals that both amplified products (TiPmk1 and TiFus3) are MAP kinase homologues isolated from *T. indica* and shows the homology with MAPKs of other fungi.

### 3.3. Influence of Host Factor(s) on Fungal Growth and Development

**Figure 2:**
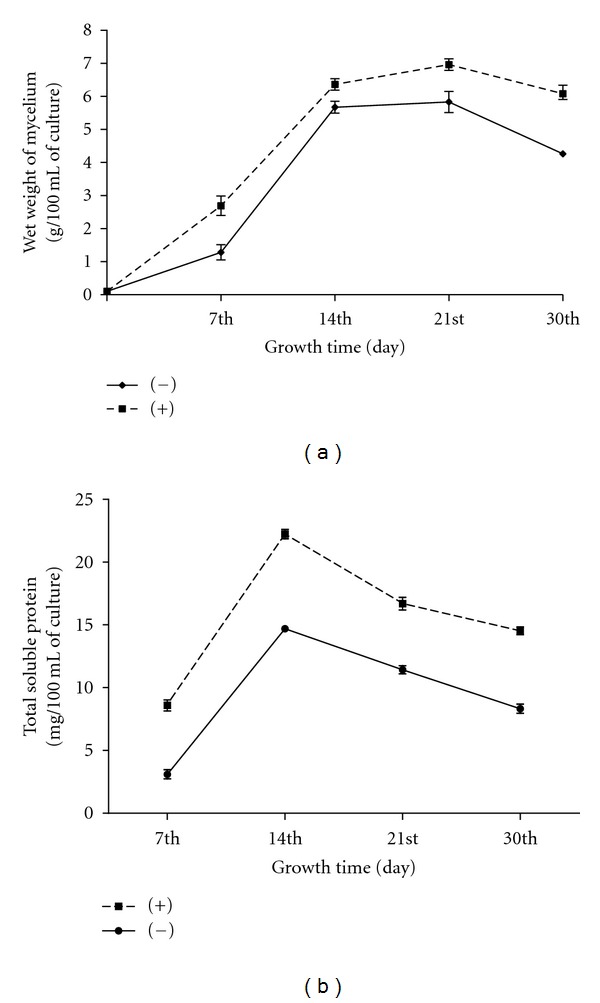
(a) Growth kinetics of *T. indica* isolate grown in presence (+) and absence (−) of host factor(s) in terms of total biomass production (g/100 mL on wet basis) in different time intervals. (b) Total soluble protein content of *T. indica *isolate grown in presence (+) and absence (−) of host factor(s) (mg/100 mL of culture) at different time intervals.

The vegetative mycelium increased exponentially (logarithmic growth phase) by lateral intercalary division up to 14 days followed by a decrease in the rate of multiplication (stationary/decline growth phase; Figure 2). The mass of mycelial mat at exponential stage was approximately 1.2 g/100 mL, and total soluble protein 3.1 mg/100 mL of culture at the 7th day rose to 5.6 g/100 mL mass of mycelial mat, and total soluble protein was 14.7 mg/100 mL of culture at the 14th day of growth cycle in absence of host factor(s) (Figures 2(a) and 2(b)); however, in presence of host factor(s), the mass of mycelia mat was 2.6 g/100 mL, and total soluble protein 8.6 mg/100 mL of culture at the 7th day rose to 6.3 g/100 mL mass of mycelia mat, and total soluble protein was 22.2 mg/100 mL of culture at the 14th day of growth cycle. After that, the decrease in growth in terms of wet mycelia weight and protein contents at the 30th day of culture both in presence and absence of host factors may be due to mycelial death on account of exhaustion of nutrients in media or transition of mycelial phase to sporogeneous phase. It was reported earlier that during sexual development of bunt fungi, fusion of compatible sporidia leads to conversion of haploid mycelial or sporidial phase to diploid teliosporic phase [37, 38]. 

These transition from mycelial to sporogeneous phase was examined at different time intervals by microscopic and haemocytometer. A clear-cut variation was observed in sporidial count as well as morphological features in fungal cultures grown at different time intervals in presence and absence of host factor(s). As shown in Table 2, host factor(s) induces the formation of mycelination which prolongs up to 21 days, while in absence of host factors, the fungal cultures undergo transition from mycelia to sporogenous phase. The mycelial growth in presence of host factor(s) was pronounced up to 21 days with intercalary division that led to thickening with multiple nuclei. In contrast, thin, long, less septet, and nucleic formation was observed in mycelial growth in absence of growth factor(s). At the 21st day of growth, there are appearances of banana-shaped sporidia which are less in the presence of host factor(s) when compared in absence of host factor(s). At 30 days, both cultures grown in presence and absence of host factors showed the transition in the developmental stages after sensing the nutritional status. At the tip of mycelia, crimpled sporogenous mycelia with formation of more banana-shaped sporidia at the tip of hyphae in absence of growth factor(s); however, in presence of growth factor(s), and the enlarged sporogenous mycelia with less formation of such sporidias were observed. The formation of few chlamydospore (immature teliospores like entities) and high number of banana-shaped allantoid sporidia (8.2 × 10^9^) were observed in fungal cultures grown in absence of host factors while no chlamydospore and comparatively less allantoid sporidia (1.3 × 10^8^) formation was observed in fungal cultures grown in presence of host factors. The influence of host factor(s) on fungal growth and development was subsequently related with stage-dependent expression of MAP kinase genes keeping in the view of investigating their role in pathogenecity and sexual development of *T. indica*.

### 3.4. Expression Analysis of MAP Kinase Genes under the Influence of Host Factor(s)

Over the past decade, it has emerged that the signal transduction pathways which regulate key virulence functions are highly conserved across a wide range of plant pathogenic fungi. In the present study, the expression of MAP kinase genes (responsible for pathogenicity in fungal systems) with respect to fungal growth and morphology was studied. Total RNA was isolated from *T. indica *cultures grown in absence and presence of host factor(s) at different time intervals (7th, 14th, 21st, and 30 day after inoculation) and isolated RNAs were subjected for two-step RT-PCR and real-time PCR using the gene-specific primers for MAPKs designated as TiFus3 and TiPmk1. As the difference in melting temperature of forward and reverse primers used in the second strand synthesis, a gradient PCR was performed for each and every combination of primers in order to decide the ideal annealing temperature for primer combinations and optimum number of PCR cycles required for discrimination of expression. It was observed that TiFus3 and TiPmk1 genes were amplified from cDNAs derived from fungal samples grown in absence and presence of host factor(s) in time-dependent manner as given below. The RT-PCR amplicons were cloned and nucleotide sequence of independent clones was determined with the dye terminator kit (ABI Prism, Perkin Elmer, NJ) and analyzed on Applied Biosystems 370 at University of Delhi. The obtained sequences are similar to the CDS sequence that we had submitted earlier in NCBI (FJ571362.1 and HQ268553).

### 3.5. Study of Expression TiFus3 under the Influence of Host Factor(s)

**Figure 3:**
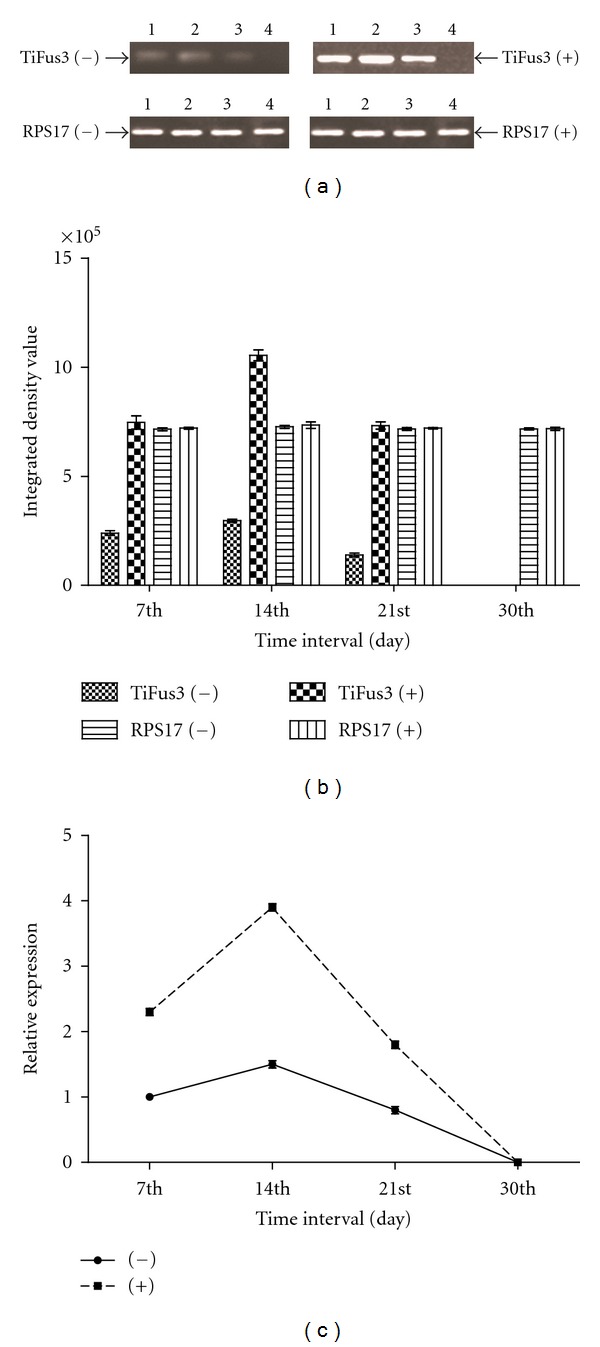
(a, b and c) Expression analysis of TiFus3 gene in *T. indica* isolate in absence of host factor (−) and in presence of host factor (+) at different days of growth and development in liquid culture. (a) Semiquantitative RT-PCR analysis—RT-PCR of expressed messenger RNA at 7th, 14th, 21st, and 30th day (lane 1 to 4, resp.) with RPS17 rRNA transcript used as internal control. (b) Densitometry analysis- Integrated density value based on densitometry analysis was done with the help of gene profiler software, Alpha Innotech Corporation USA. (c) Quantitative real-time PCR analysis—relative expression using real-time PCR.

For the second strand synthesis of TiFus3 gene, annealing temperature for PCR amplification was optimal to 57°C as determined by the gradient PCR. A 180 bp band was detected after the amplification of cDNA by RT-PCR analysis which showed higher expression of TiFus3 transcripts in fungal cultures grown in presence of host factors while comparing with control (absence of host factors). In the case of positive control, a 210 bp band of RPS17 was amplified. No amplification was detected in negative control which was carried out without cDNA indicating no DNA or mRNA contamination in reagents used in the amplification. For the time-dependent expression of Fus3 transcripts, KB fungal cultures grown in absence and presence of host factors at the 7th, 14th, 21st, and 30th day were used in the study. For semiquantitative RT-PCR, densitometry (relative values) and real-time PCR clearly showed that RPS17 gene expression was constitutive, that is, similar expression of RPS17 gene, whereas Fus3 mRNA transcripts were differentially expressed at all time intervals in both host factors treated and untreated fungal cultures (Figures 3(a) and 3(b)). The expression profiling and densitometry analysis of TiFus3 gene showed gradual increase in amplification up to 14 days and henceforth a reduction was noticed with no amplification at 30th day. The results of RT-PCR and real-time PCR was almost parallel to each other.

For reverse transcription efficiency in real-time PCR, reaction conditions were optimized with endogenous control (RPS17) and TiFus3 genes. Different dilutions of cDNA were used and based on *C*_*T*_ values, these efficiencies were almost equal. This showed that RPS17 gene can be used as endogenous control to analyze the relative expression of TiFus3 gene at different time interval. Quantitation of the TiFus3 transcripts was done in fungal cultures grown in absence and presence of host factors at the 7th, 14th, 21st, and 30th days Relative expression of Fus3 was calculated (Figure 3(c)) and it was expressed at significantly higher level (1.5 folds) at 14th day, followed by 21st day (0.8 fold), and 30th day (0.0, no expression) as compared to 7th day of cultures grown in absence of host factors. However, comparing the fungal cultures grown in presence of host factors, it was found that TiFus3 gene was expressed at significantly higher level at the 7th day (2.3 folds), 14th day (3.9 folds), 21st day (1.8 folds), and 30th day no expression was detected. The expression of TiFus3 gene was positively correlated with wet mycelia weight and total soluble protein in the presence (*r* = 0.0713 and 0.4581) and in the absence of host factor(s) (*r* = 0.0991 and 0.3610), respectively.

Components of three MAP kinase pathways have been identified by genome sequence analysis in the filamentous fungus *Neurosphora crassa*. One of the predicted MAPK in *N. crassa, *MAK-2, shows similarity to Fus3p and Kss1p of *Saccharomyces cereviceae*, which are involved in sexual reproduction (mating type switching) and filamentation, respectively [39]. Participation in multiple MAPKs is a characteristic of the MAPK signaling pathways of *S. cereviceae*, where Fus3p is specific to the pheromone-induced mating MAPK pathway, while other components of this pathway also participate in the MAPK pathway which controls filamentous growth [24]. MAP kinase signaling components (Fus3) might regulate downstream transcription factors gene and transcription factor gene shows the transcriptional changes with disease development or penetration, dimorphic switch in the presence of pheromone. In the present study, Fus3 gene expression has been noticed in inducible manner by host factor(s) and the gene expression is very negligible and almost constitutive in control (absence of host factors). Host factor(s) treatment tends to increase the mycelination in fungal cultures by lowering the sporidial production. Hence, it can be concluded that increase in mycelination of fungi leads to impose more pathogenicity levels in the host and prolific multiplication of pathogen inside host leading to more damage to developing grains. These findings reveal that the host factor(s) provide an environment for induction of TiFus3 gene expression and in turn might promote morphogenetic development through mating type switching and induced mycelination under the influence of host factors.

### 3.6. Study of Expression TiPmk1 under the Influence of Host Factor(s)

In *Magnaporthe grisea*, a well-conserved mitogen-activated protein (MAP) kinase gene Pmk1 is essential for fungal pathogenesis. Zheng et al. [21] tested whether the same MAP kinase is essential for plant infection in the gray mold fungus *Botrytis cinerea*, a necrotrophic pathogen that employs infection mechanisms different from those of *M. grisea.* They used a polymerase chain reaction-based approach to isolate MAP kinase homologues from *B. cinerea*, the *Botrytis* MAP kinase required for pathogenesis (BMP). MAP kinase gene was highly homologous to the *M. grisea* Pmk1, Bmp1 is a single-copy gene. Bmp1 gene replacement mutants produced normal conidia and mycelia but were reduced in growth rate on nutrient-rich medium. Bmp1 mutants were nonpathogenic on carnation flowers and tomato leaves. Reintroduction of the wild-type Bmp1 allele into the Bmp1 mutant restored both normal growth rate and pathogenicity. Further studies indicated that conidia from Bmp1 mutants germinated on plant surfaces but failed to penetrate and macerate plant tissues, Bmp1 mutants also appeared to be defective in infecting through wounds. These results indicated that Bmp1 is essential for plant infection in *B. cinerea*, and this MAP kinase pathway may be widely conserved in pathogenic fungi for regulating infection processes. However, under *in vitro* cell culture system, host factors might regulate MAP kinase signaling components (Pmk1) and its downstream transcription factor genes which led the transcriptional changes with invasive growth, cell wall integrity, and spore wall assembly.

**Figure 4:**
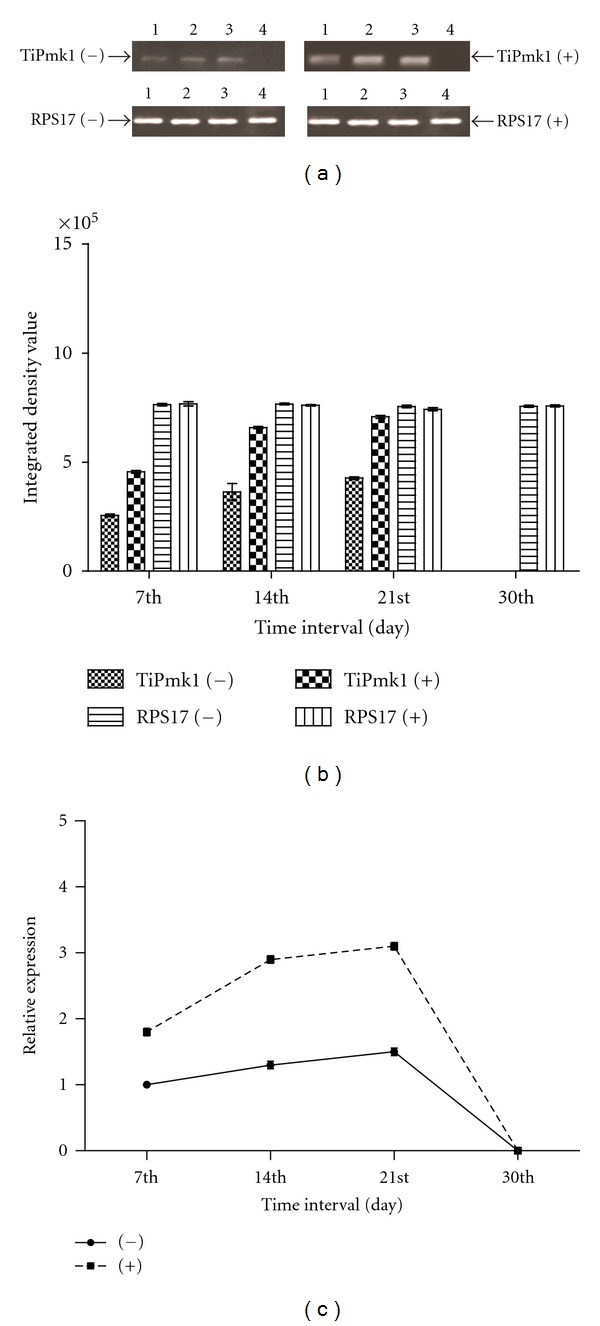
(a, b and c) Expression analysis of TiPmk1 gene in *T. indica* isolate in absence of host factor (−) and in presence of host factor (+) at different days of growth and development in liquid culture. (a) Semiquantitative RT-PCR analysis—RT-PCR of expressed messenger RNA at the 7th, 14th, 21st, and 30th day (lane 1 to 4, resp.) with RPS17 rRNA transcript used as internal control. (b) Densitometry analysis-integrated density value based on densitometry analysis was done with the help of gene profiler software, Alpha Innotech Corporation USA. (c) Quantitative real-time PCR analysis—relative expression using real-time PCR.

With respect to the cDNA amplification of TiPmk1 gene, 57°C was used as the annealing temperature. Samples of cDNA prepared from fungal culture grown in absence and presence of host factors were subjected for second strand synthesis of semiquantitative RT-PCR and quantitative real-time PCR. RT-PCR results showed amplification of an amplicon 210 bp in size in both types of samples. However, the expression of Pmk1 was higher in fungal cultures grown in presence of host factors when compared with its control (absence of host factors). In this case positive control (RPS17 gene) amplified 210 bp band from cDNA isolated from both types of fungal cultures. In order to determine the change in time-dependent expression of Pmk1, fungal cultures grown in absence and presence of host factors were harvested at the 7th, 14th, 21st, and 30th day in cultures. Gradual increase in amplification was detected up to the 21st day and thereafter, a decline was observed and with no amplification at the 30th day. The expression profiling and densitometry analysis of TiPMK1 gene in absence and presence of host factors are given in Figures 4(a) and 4(b). No amplification was detected in negative control which was carried out without cDNA indicating no DNA or mRNA contamination in reagents used in the amplification.

The results of reverse transcription and real-time PCR were almost parallel to each other and also observed in case of TiFus3 gene. Quantitation of the TiPmk1 transcripts expressed in fungal cultures grown in absence and presence of host factors at different days (7th, 14th, 21st, and 30th day) was noticed. Relative expression of TiPmk1 was calculated in both types of fungal cultures (Figure 4(c)) and it was expressed at significantly higher level in the 21st day (1.4 folds) followed by 14th (1.3 folds), 30th (0.0, no expression) as compared to the 7th day of cultures grown in absence of host factors. However, comparing the fungal cultures grown in presence of host factors, it was found that TiPMK1 gene was expressed at significantly higher level at the 7th day (1.8 folds), 14th day (2.9 folds), 21st day (3.1 folds), and 30th day (0.0 fold, no expression was detected). The expression of TiPmk1 gene was positively correlated with wet mycelia weight and total soluble protein in the presence (*r* = 0.2179 and 0.4477) and in the absence of host factor(s) (*r* = 0.2868 and 0.3740), respectively.

Such results clearly revealed the higher expression of TiPmk1 gene in presence of host factors and in turn induction of pathogenecity. It is also evident from the results of pathogenecity testing under the influence of host factors in two varieties of wheat differing in their resistance to KB. The percentage of disease scoring was increased (percentage of infection from 23.6 to 35.5 in susceptible and from 17.7 to 21.6 in resistant varieties in Table 1) by the fungal culture grown in presence of host factors. It might be possible that the host-pathogen interaction requires mechanism to detect suitable signals from host that triggers a signal transduction cascade in fungal pathogen and that induce the expression of appropriate virulence factors, as has been well characterized in numerous other fungal-plant-pathogen interaction [22].

The above findings related to the expression of MAP kinase genes indicate the clear involvement of MAPK signal transduction cascade in development of pathogenesis in *T. indica.* In these perspectives, it is appropriate to study the expression of genes of MAPK module which will provide strong clues in order to unveil the molecular mechanism associated with fungal development and pathogenesis of *T. indica. *The expression of two genes belongs to MAPK (TiFus3 and TiPmk1) were detected by RT-PCR and real-time PCR in *T. indica*. In the present study, we found the amplification of TiFus3 and TiPmk1 gene by gene-specific primer at different time interval and also found expression increase in presence of host factor(s) in *T. indica *when compared in absence of host factor(s). It supports a causal link between host factors and MAP kinase function in influencing the disease progression and pathogenesis during host-pathogen interaction. With the present observations, it can be indirectly interpreted the involvement of mating type switching (TiFus3) as well as pathogenicity (TiPmk1) subpathways of fungal MAPK signal transduction cascade in the morphogenetic development and pathogenesis of *T.indica*. Remarkably, we have further shown the role of such orthologues of TiFus3 and TiPmk1 in *T. indica* that are also present in *Magnaporthe grisea* and *S. cereviceae. *Identification of downstream MAP kinase regulated transcription factors, most importantly those that regulate the critical penetration and colonization, would open the door to a broader understanding of the nature of the regulatory networks which govern pathogenic development and why certain signaling pathways are universally essential for pathogenicity. Ultimately, this may will lead to the identification of potential novel fungicides hitting such key targets of MAP kinase machinery which contribute significantly to the fungal growth and pathogenecity. Hence, delineation of signal transduction machinery is very much crucial in framing biotechnological control measures against such pathogens.

## Figures and Tables

**Table 1:** Pathogenicity testing of two varieties under different treatments on the basis of disease scoring after the crop harvest.

Treatments	Total number of seeds	Number of seeds with susceptible reaction	Percentage infection	Coefficient of infection in %	Overall response value***
**(1)**	**(2)**	**(3)**	**(4)**	**(5)**	**(6)**
*Resistant*					
RC	116	0	0	0	0
RP	85	15 (14* + 1**)	17.65	0.04	10.59^#^
RHFP	88	19 (17* + 2**)	21.59	0.05	20.51^#^
*Susceptible*					
SC	109	0	0	0	0
SP	89	21 (11* + 10**)	23.6	0.07	34.69^#^
SHFP	90	32 (17* + 15**)	35.55	0.12	136.51^#^

*t* = 0.3172.

^#^ *P* < 0.05.

*Incipient infection.

**Blackening of seed upto 1/2.

***Products of numerical values in column 3, 4, 5.

**Table 2:** Determination of morphological variation and sporidial count of *T. indica* isolate grown in presence and absence of host factor(s).

Days of culture	In absence of host factor(s)		In presence of host factor(s)	
	Morphological variation	Sporidial count (Number of sporidia/mL)	Morphological variation	Sporidial count (number of sporidia/mL)
7th	Thin long, interwoven vegetative mycelia with less septation and nuclei	Nil	Thick, comparatively small vegetative mycelia with extensive septation and multiple nuclei	Nil

14th	Thickened, branched mycelia which produces secondary sporidia at the tip of lateral small branches	1.8 × 10^3^	Thickened, small vegetative mycelia	Nil

21st	Acicular, tapered, banana-shaped high number sporidia and sporogenous mycelia seen occasionally	3.9 × 10^9^	Thickened mycelia which produce few banana-shaped allantoid sporidia	4.1 × 10^7^

30th	Crumpled sporogenous mycelia with short sporogenous hypal branches, terminal/intercalary rounding up of hypal cells, sometimes in chain. Few small chlamydospore like structures	8.2 × 10^9^	Enlarged and many sporogenous mycelia and less hyphal branches and not much chlamydospore formation	1.3 × 10^8^
